# Application of Plasma Exchange in Patients with History of Unexplained Recurrent Abortion: A Case Series

**Published:** 2013-03-06

**Authors:** Mohammad Ali Roghaei, Farzane Jamdar, Azadeh Ghaheri

**Affiliations:** 1 Department of Obstetrics and Gynecology, School of Medicine, Isfahan University of Medical Sciences, Isfahan, Iran; 2 Department of Epidemiology and Reproductive Health at Reproductive Epidemiology Research Center, Royan Institute for Reproductive Biomedicine, ACECR, Tehran, Iran

**Keywords:** Recurrent Abortion, P-Incompatibility, Plasma Exchange, Anti-P Antibody

## Abstract

**Background:**

Immune-mediated recurrent pregnancy loss (RPL) has received more attention than any other single etiologic classification. Individuals with rare blood group P have an antipp1pk antibody in their serum, which causes recurrent abortion in the early stages.

**Materials and Methods:**

In this case series study, 11 patients with unexplained RPL who had anti-P antibody in their serum were treated by plasma exchange during their next pregnancies. To evaluate the efficacy of the treatment, we monitored fetal development using ultrasonography and intensive prenatal care. All calculations were performed with the SPSS version 16.

**Results:**

All patients who were treated by plasma exchange progressed to live birth. The mean gestational age at the time of termination was 37.5 ± 0.69 weeks. The mean weight of the newborns was 2729.09 ± 389.88 g. None of the newborns required exchange transfusion.

**Conclusion:**

P-incompatibility is one rare but important cause of unexplained RPL and also a basis for therapeutic intervention via early antibody removal by plasma exchange.

## Introduction

Spontaneous abortion is one of the most common complications of pregnancy. Traditionally, recurrent pregnancy loss has been defined as the occurrence of 3 or more abortions before 20 weeks. Etiologies of RPL consist of genetic factors, thrombophilias, anatomic abnormalities, endocrine abnormalities, maternal infections and immunologic factors. Even after a thorough evaluation, however, the potential cause remains unexplained in about one third to one half of cases. Immune-mediated RPL has received more attention than any other single etiologic reason, nevertheless, techniques for diagnosis and treatment of most cases remain unclear (1).

The P blood group system which was first reported by Landsteiner and Levin in 1927 is another group of carbohydrate antigens controlled by a specific glycosyl transferase. Its clinical significance is in rare cases of syphilis and viral infections and early abortion. The P blood group consists of 5 phenotypes p1, p2, p1k, p2k and P as shown in table 1 (2, 3).

A rare phenotype of system that lacks either P1 or Pk antigen is denoted as small-p type. The prevalence of the phenotype has been reported to be 1/10000-1/100000 (2,3). Individuals with this phenotype have an antibody cocktail that includes anti-p, p1 and Pk as natural antibodies (2).The presence of PP1Pk antibody is considered a main cause of repeated spontaneous abortion in early pregnancy and hemolytic disease in newborns (4-9).

**Table 1:** Properties of respective P-system blood types


			Prevalence	
Phenotype	Antigens on RBC	Antibodies in serum	Japanese	Caucasian

**P1**	P1, P	None	35%	75%
**P2**	P	Anti-P1 antibody	65%	25%
**P1K**	P1, PK	Anti-P antibody	Rare	Rare
**P2K**	PK	Anti- P antibody	Rare	Rare
**P**	None	Anti-PP1PK antibody	1/10000 -1/100000	1/10000 -1/100000


The fetus expresses antigens on trophoblastic tissues as early as 3 weeks (2). The maternal antibodies directed against these antigens have been associated with abortion in more than half of the affected pregnancies, as well as fetal growth retardation in ongoing pregnancies; many studies suggest that early plasma exchange and lowering antibody level in these women could be therapeutic (2-4, 10, 11). Plasma exchange is an extracorporeal blood purification technique for removal of large molecular weight substances (e.g. antibodies, paraproteins) from the plasma (12). The aim of this study is to evaluate the efficacy of treatment by plasma exchange in patients with rare blood group p.

## Materials and Methods

In this case series study, we report 12 cases with P-blood type of which 11 of them were treated successfully with antibody removal therapy between 2007-2012 in Al Zahra Hospital, Isfahan, Iran. The study was approved by the Department of Ethics at Isfahan University of Medical Sciences.

These patients had history of recurrent pregnancy loss. Anatomic, genetic, endocrine and thrombophilia disorders were excluded in them. Chromosome abnormalities were excluded by parental peripheral blood karyotyping with banding techniques. Anatomic disorders were excluded by hysterosalpingography and to exclude the endocrine and thrombophilia disorders, patients were tested for CBC, diff, Protein S&C, Lupus anticoagulant testing, Anticardiolipin antibody, TSH, T4, Anti TPO, Anti TGB, FBS, GCT, serum homocysteine levels, Anti β2glycoprotein 1, Anti thrombin III, Factor V Leiden, ANA, ANCA and APCA (anti paternal complement antibody). These patients had been assessed for atypical antibodies and anti- P antibody in their serum. For assessing the anti- Pantibody our kit was Biotestcell-P3 (Ref 816017) (Bio-Rad Medical Diagnostics, Germany). The test principle is a hemagglutination test. Anti-p antibody was positive in these patients. Patients were consulted about treatment by plasma exchange and if they became pregnant, intensive prenatal care and plasma exchange was initiated. Written informed consent was obtained from all participants.

Therapeutic plasma exchange was carried out twice a week from early stages of pregnancy (if pregnancy tests were positive) in AL Zahra Hospital. In every course about 900 cc of patient’s blood was taken. Plasma exchange was performed with centrifugation devices used in blood banking procedures and antibodies were separated from blood cells and removed. Then the residual blood was reinfused to the patients. We used normal saline serum and 5 or 20% albumin solution as a substitution fluid. Plasma exchange procedure was performed up to termination of the pregnancy. To evaluate the efficacy of treatment, we monitored fetal development using ultrasonography. Considering the rarity of the maternal blood type and the safety of the fetus, we decided to carry out the scheduled delivery by cesarean.

All calculations were performed with the SPSS Version 16.0 for Windows (SPSS Inc., Chicago, Illinois, USA).

## Results

In this study we successfully treated 11from 12 patients with history of unexplained recurrent abortion with plasma exchange. Patient descriptive statistics are presented in table 2. One of the patients did not consent to participate and was excluded. She was a 29 year old woman, who had history of 2 previous spontaneous abortions with no live birth. Her blood group and rh was B positive.

The relative frequency of primary RPL was 83.3% and the relative frequency of secondary RPL was 16.8% in patients. All previous abortions had happened in early stages of pregnancies, specifically in the first trimester. None of the patients had history of blood transfusion. One of the patient’s RH was negative and the other 11 were positive.

All of the p-positive patients treated by plasma exchange had successful pregnancy proceeding to full-term gestational age and healthy newborns. The plasma exchange procedure was performed 59.18 ± 14.1times (range of 20-75 times) on average during the pregnancy. All patients continued plasma exchange up to termination time, except one patient who could not continue the treatment because of far distance to AL Zahra Hospital.

As shown in table 2, three of the newborns (27.3%) weighed below 2500 g but in these newborns the weight was also higher than 2100 g. None of them had anemia or severe jaundice. None required exchange transfusion.

**Table 2:** Descriptive statistics of patients


	Age (Y)	Number of gravidities	Number of gbortions	Number of children being born alive	Blood group &Rh	Gestational age at the time of termination (week)	Birth weight of newborn (g)

**Case 1**	26	4	3	1	O^+^	38	3070
**Case 2**	26	5	4	1	A^+^	37	3300
**Case 3**	26	5	5	0	O^+^	38	2550
**Case 4**	28	5	5	0	B^+^	38	2800
**Case 5**	28	7	7	0	AB^+^	39	2400
**Case 6**	34	8	8	0	A^+^	38	3000
**Case 8**	32	6	6	0	O^+^	37	2300
**Case 9**	32	5	5	0	B^+^	37	3300
**Case 9**	19	2	2	0	B-	37	2600
**Case 10**	33	4	4	0	A^+^	37	2500
**Case 11**	29	2	2	0	O^+^	37	2200
Total*	28.45 (4.30)	5 (2-8)	5 (2-8)	0 (0-1)	(O^+^)	37.54 (0.69)	2729.09 (389.88)


*Mean (SD) for quantitative variables, Median (Range) and (Mode) for qualitative variables.

## Discussion

Approximately 70% of human pregnancies fail to achieve viability and an estimated 50% are lost before the first missed menstrual period. Recurrent abortion has been defined as the occurrence of 3 or more clinically recognized pregnancy losses before 20 weeks (1). Some authors suggest that clinical investigation of pregnancy loss, however, may be initiated after two consecutive spontaneous abortions.

Maternal immune recognition and response undoubtedly plays an important role in normal pregnancy and alloimmune disorders may be a cause of otherwise unexplained recurrent pregnancy loss (RPL). Alloimmune disorders involve an abnormal maternal immune response to paternally derived antigens on embryonic tissues; possibilities include maternal cytotoxic antibodies, absent maternal blocking antibodies and disturbance in natural killer cell function and distribution (13).

The presence of PP1Pk antibody is considered a main cause of repeated spontaneous abortion in early pregnancy and hemolytic disease in new borns (4-7) but which component is responsible for RPL is not clear yet (4). Some previous studies suggested that anti-p component of anti-pp1pk is responsible for abortion in P women (14, 15). Many studies suggested that plasmapheresis is a successful treatment in P-incompatibility pregnancies (2-4, 6, 10, 11). Abortions mainly occur in the first-trimester and all fetuses that remain alive in this critical period will develop to healthy newborns (4).

The timing of the initiating is particularly important because P and Pk antigens are expressed as early as 3 weeks of age on the trophoblastic tissues. Fetal tissue begins to express P1 antigen at week 19 or later. The anti-P1 antibody is not as pathogenic as the anti-P and Pk antibodies. The P1 antigen is reported to inhibit expression of P and Pk antigen (2). Antibody removal therapy is needed from the early stages, to keep the antibody-titer low to maintain normal placenta functions. Conversely the duration of therapy is controversial, especially at week 20 or later (2, 3).

Some studies suggest that the frequency of plasmaphresis should be lowered and gradually tapered at 16 weeks and thereafter. Also in our study one of the patient who could not continue the treatment up to term, (plasma exchange performed up to week 20) had a successful pregnancy and living child. For the others we continued therapy to ensure the safety of the fetus because no adverse effect of plasma exchange was observed, and because the precise mechanism and the effect of antip antibody has not yet been elucidated (2, 3).

More than 70% of newborns weighed more than 2500 g and pathologic intrauterine fetal growth restriction was not detected. No anemia nor severe jaundice was observed and transfusion was unnecessary. So we successfully managed P-incompatibility pregnancies by using plasma exchange.

This is the first study in Iran that tried to evaluate the efficacy of treatment by plasma exchange in patients with rare blood group P. It seems that more studies are needed about the P phenotype.

## Conclusion

P-incompatibility is one rare but curable causes of unexplained RPL and early intense antibody removal could be therapeutic. For therapeutic intervention plasma exchange is efficacious and suitable modality.
